# Five key factors determining pairwise correlations in visual cortex

**DOI:** 10.1152/jn.00094.2015

**Published:** 2015-05-27

**Authors:** David P. A. Schulz, Maneesh Sahani, Matteo Carandini

**Affiliations:** ^1^COMPLeX, London, United Kingdom;; ^2^Gatsby Computational Neuroscience Unit, London, United Kingdom; and; ^3^Institute of Ophthalmology, University College London, London, United Kingdom

**Keywords:** variability, sensory cortex, spontaneous activity, natural stimuli, functional connectivity

## Abstract

The responses of cortical neurons to repeated presentation of a stimulus are highly variable, yet correlated. These “noise correlations” reflect a low-dimensional structure of population dynamics. Here, we examine noise correlations in 22,705 pairs of neurons in primary visual cortex (V1) of anesthetized cats, during ongoing activity and in response to artificial and natural visual stimuli. We measured how noise correlations depend on 11 factors. Because these factors are themselves not independent, we distinguished their influences using a nonlinear additive model. The model revealed that five key factors play a predominant role in determining pairwise correlations. Two of these are distance in cortex and difference in sensory tuning: these are known to decrease correlation. A third factor is firing rate: confirming most earlier observations, it markedly increased pairwise correlations. A fourth factor is spike width: cells with a broad spike were more strongly correlated amongst each other. A fifth factor is spike isolation: neurons with worse isolation were more correlated, even if they were recorded on different electrodes. For pairs of neurons with poor isolation, this last factor was the main determinant of correlations. These results were generally independent of stimulus type and timescale of analysis, but there were exceptions. For instance, pairwise correlations depended on difference in orientation tuning more during responses to gratings than to natural stimuli. These results consolidate disjoint observations in a vast literature on pairwise correlations and point towards regularities of population coding in sensory cortex.

the firing of different cortical neurons is often coordinated, sometimes over large distances and even across different areas (Volgushev et al. 2011; Hipp et al. 2012). Although such coordination may be quantified by sophisticated techniques such as generalized-linear models (Truccolo et al. 2005) or information theory (Gawne et al. 1996), the most widespread measure has been the Pearson correlation coefficient computed between synchronous spike counts of neurons (Perkel et al. 1967; Bair et al. 2001; Cohen and Kohn 2011). Pairwise correlations arise because neurons have overlapping stimulus selectivity. These stimulus-driven (“signal”) correlations are explained straightforwardly. More intriguing are the correlations in the variable firing rate measured when the same stimulus is repeated multiple times, known as “noise correlations.”

Noise correlations play an important role in neuroscience. They help determine the representational capacity of a neural code (Zohary et al. 1994; Abbott and Dayan 1999; Sompolinsky et al. 2001; Averbeck et al. 2006), they aid inferences about anatomical connectivity (Toyama et al. 1981b; Ts'o et al. 1986; Alonso and Martinez 1998; Ko et al. 2011), and they constrain models of cortical circuitry and network dynamics (Ginzburg and Sompolinsky 1994). The impact of pairwise correlations on neuronal networks increases with the size of the population considered (Averbeck et al. 2006; Schneidman et al. 2006), so even weak correlations may have profound consequences.

A debate has centered on the strength of noise correlations, especially in sensory areas such as primary visual cortex (V1). An influential study in area V1 reported extremely small average noise correlations, in the order of 0.001 (Ecker et al. 2010), matching similar measurements in auditory cortex (Renart et al. 2010). These results contrast with earlier studies from multiple laboratories, which had found average noise correlations in area V1 to be much larger at ∼0.1 (Benucci et al. 2013; Schölvinck et al. 2015), ∼0.20 (Kohn and Smith 2005), 0.18 (Smith and Kohn 2008), ∼0.25 (Reich et al. 2001), or even ∼0.35 (Gutnisky and Dragoi 2008; see Cohen and Kohn 2011 for an extensive review). The sources of this discrepancy remain unclear.

A possible source of discrepancy lies in the firing rate evoked by the stimuli. Pairwise correlations are widely believed to increase with firing rate. This effect has been seen in simulations (Dorn and Ringach 2003; de la Rocha et al. 2007) and in vitro (de la Rocha et al. 2007). Moreover, firing rate has been seen to increase noise correlations in area MT (Bair et al. 2001) and V4 (Cohen and Maunsell 2009) and is a plausible determinant of correlations measured across studies (Cohen and Kohn 2011). However, this relationship has not been consistently observed in all studies performed in vivo. Some found no dependence, either at the level of populations or at the level of individual neurons (Kohn and Smith 2005), or only weak dependence, mostly due to slow covariations (Ecker et al. 2010).

Another possible source of discrepancy lies in the quality of spike isolation. It has been proposed that correlations depend on spike isolation (Ecker et al. 2010) and are inflated by falsely assigning spurious spikes during spike sorting. However, the implications of this effect go both ways, as excessive criteria in spike isolation would make one drop legitimate spikes and thus underestimate correlations (Cohen and Kohn 2011).

To assess the impact of these and other factors, we considered a set of correlations measured in over 22,705 pairs of V1 neurons of anesthetized cats. We studied how they depended on a set of 11 physiological and functional factors during the presentation of natural stimuli, artificial stimuli (flashed static gratings and drifting gratings), and blank screens (spontaneous activity). Because these factors are themselves not independent, we untangled their influences using a nonlinear additive model. The model revealed a subset of five key factors that play a predominant role in determining pairwise correlations.

## MATERIALS AND METHODS

The data used for this study were acquired for studies that have been published (Benucci et al. 2007, 2009). All experiments were approved by the Institutional Animal Care and Use Committees of the Smith-Kettlewell Eye Research Institute and conducted according to the *Guidelines for the Care and Use of Mammals in Neuroscience and Behavioral Research* from the National Institutes of Health.

### 

#### Surgical procedure.

We analyzed data obtained from seven female adult cats (2–4 kg) following surgical procedures that have been described in detail (Benucci et al. 2007, 2009). Briefly, cats were anesthetized first with ketamine and xylazine and then with sodium pentothal and fentanyl, supplemented with inhalation of N_2_O. A craniotomy was performed over area V1 (usually area 18, occasionally area 17). The eyes were treated with topical atropine and phenylephrine and protected with contact lenses. A neuromuscular blocker was given to prevent eye movements (pancuronium bromide). The animal was artificially respirated and received periodic doses of an antibiotic (cephazolin), an antiedematic steroid (dexamethasone), and an anticholinergic agent (atropine sulfate). Fluid balance was maintained by intravenous infusion. The level of anesthesia was monitored through the EEG. Additional physiological parameters that were monitored include temperature, heart rate, end-tidal CO_2_, and lung pressure. Experiments typically lasted 48–72 h.

#### Stimuli.

**Fig. 1.**
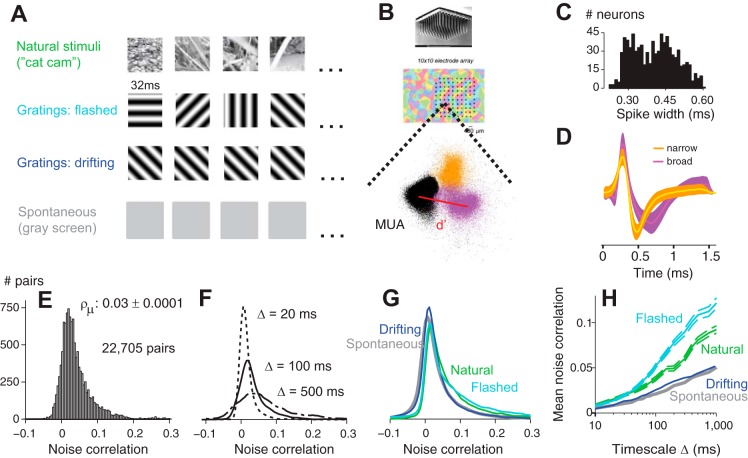
Experimental setup and distribution of noise correlations. *A*: 4 types of stimuli, with illustrative single frames. *B*: Utah array and the position of its sites in an orientation preference map of cat V1. On each electrode, spike waveforms were clustered in principal component analysis space. Two clusters correspond to single units (orange and purple) and one to multiunit noise [multiunit activity (MUA), black]. *C*: histogram of spike widths shows 2 clear distinct peaks, corresponding to narrow-spiking and broad-spiking cells. *D*: waveforms of a narrow-spiking cell (orange) and a broad-spiking cell (purple). They correspond to the colored clusters in *B*. *E*: distribution of pairwise noise correlations at a timescale of Δ = 100 ms obtained across all cats (*n* = 7) and all stimulus conditions. *F*, Same as in *E*, but computed at 3 timescales (Δ = 20, 100, and 500 ms). *G*: same as in *E* but split into each stimulus condition. *H*: average noise correlations as a function of timescale for each stimulus condition. Error bars are included for only 2 conditions for ease of visualization but are comparable across conditions.

Four types of stimuli were employed: natural stimuli, flashed gratings, drifting gratings, and blank (gray) screens (Fig. 1*A*). Grating stimuli were full-field (40 × 40°), presented monocularly on a CRT monitor (Sony Trinitron 500PS, refresh rate of 125 Hz, mean luminance of 32 cd/m^2^). Gratings had one of 8 or 16 equally spaced orientations in the range from 0 to 180°. Flashed gratings were presented statically for four video frames (4 × 8 ms = 32 ms) in random orientations and spatial phases. Drifting gratings, instead, maintained the same orientation for several seconds, while drifting at 5 Hz. Spatial frequencies varied from 0.2 to 0.4 cycles/degree. The contrast varied between experiments but was typically high (50–100%). Stimuli were preceded by 2 s of uniform gray, typically lasted ∼10 s, and were presented in random order in blocks, each typically presented 10–20 times. Natural stimuli were movies lasting ∼10 s and presented ∼10 times. One movie was captured by attaching a small video camera to a cat's head and allowing it to roam freely in the woods (“Cat cam”; Betsch et al. 2004). The second movie contained 10-s sequences of an animated cartoon (“Tarzan”). In each block of stimulus presentations, we also presented a blank stimulus (gray screen) of the same duration, to measure spontaneous activity.

#### Spike sorting.

Neural signals were recorded using a 96-channel silicon Utah probe (Blackrock, Salt Lake City, UT). Each time the signal on a channel exceeded a threshold set manually before the session, a 1.6-ms-long snippet (48 samples) was stored as the putative spike waveform vector. Thus spikes <1.6 ms apart on a single channel could not be resolved. This limitation was not critical in our data as neurons fired sparsely and such overlaps were rare. Near-coincident spikes on different channels could still be resolved.

We used principal component analysis of each electrode's waveforms to identify a five-dimensional subspace of greatest variation. We projected the waveforms into this space, and we fitted a mixture of Gaussians to the data using KlustaKwik (Harris et al. 2000) (Fig. 1*B*). Clusters were manually checked using custom-made software programmed in Matlab, discarding clusters with nonphysiological waveform shapes and merging clusters likely to belong to the same neuron based on the interspike intervals and on the cross-correlogram. Rarely, clusters contained spikes occurring only in the beginning of a session; we discarded these as they were likely to represent neurons that dropped out of the recording. Finally, we discarded clusters where the waveforms changed shape progressively in time. Such drifts, however, were rarely observed, perhaps due to the stabilizing effects of the array. After taking all these measures to ensure good spike sorting, we were left with a sample of 843 neurons in 7 cats.

The accuracy of spike sorting can be measured only when ground truth is available (Harris et al. 2000), so we settled on an estimate of spike isolation quality: the distance from each single unit cluster to the “multiunit” cluster, which typically contained hundreds of thousands of unsorted, low-amplitude spikes. We first projected the distributions from the five-dimensional principal component analysis space onto the vector joining the mean of the multiunit distribution to the mean of the single unit distribution (Fig. 1*B*), which yielded two one-dimensional distributions. These distributions were roughly Gaussian, and we computed the distance between their means divided by the geometric mean of their standard deviations. This measure is known as d′ (d-prime) or “isolation distance” (Nauhaus et al. 2008).

#### Noise correlations.

We measured correlations using spike trains binned with precision Δ collected from *N* stimulus repetitions (trials). To study the structure of noise correlations at different timescales, we varied the bin width from 10 to 1,000 ms.

In principle, noise correlation is measured by computing the average across trials μ_*x*_, μ_*y*_ of the firing rates of two neurons, subtracting it from the measured firing rates, and calculating the Pearson correlation coefficient from the residuals. An equivalent calculation involves estimating the noise covariance by computing the covariance of two responses, subtracting from that the covariance between the two trial-averaged responses, (μ_*x*_, μ_*y*_), and then dividing this noise covariance by the geometric mean of the noise variances.

However, these procedures are correct only if one has a large number of trials. With fewer trials, the trial-averaged response will retain some noise and lead to bias. Specifically, if the noise in a single trial has variance σ^2^, then the average of *N* independent responses will retain a residual noise level with variance $\frac{\sigma^{2}}{N}$. This noise will inflate estimates of signal variance, at the expense of estimates of noise variance (which is the difference of total variance and signal variance). Because noise variance appears in the denominator when computing noise correlations, underestimating it biases noise correlations upwards.

As relatively few trials were available (typically <20), we chose an estimator of noise correlation which was resistant to bias. This estimator operates on binned spike trains and is approximately equivalent to integrating the cross- and autocorrelograms of the two cells under windows proportional to bin size (for derivation see appendix).

#### Factors.

We considered the influence on pairwise correlations of 11 factors.

*1*) Cortical distance. The distance between the electrodes from which two neurons were recorded. The closest neighboring electrodes were 400 μm apart. Units recorded from the same electrode were not included.

*2*) Tuning distance. The absolute difference in preferred orientation between two neurons. The orientation tuning curve of each neuron in response to gratings was estimated by fitting a circular Gaussian to the responses. Only well-tuned neurons were considered for this analysis. Since orientations range from 0 to 180°, tuning distance ranges from 0 to 90°.

*3*) Firing rate. The firing rate of a neuron averaged over time during presentation of a visual stimulus or during spontaneous activity. Only neurons with a minimum firing rate of 0.2 spikes/s were considered in this study.

*4*) Spike width. The width of a neuron's averaged spike waveform, defined as the time between the peak and trough. This measure distinguished two groups of neurons, with broad and narrow spikes (Fig. 1, *C* and *D*).

*5*) Spike isolation. The d′ measure of spike isolation defined above.

*6*) Tuning width. The width of the orientation tuning curve obtained at the points where the response was half of its peak. Only well-tuned neurons were considered in this analysis.

*7*) Latency. Defined as the time to the peak of the neuron's response to an optimally oriented grating, determined by spike-triggered averaging of the flashed-grating stimulus.

*8*) Linearity. The ratio of the first harmonic (F1 component) and the mean (F0 component) in response to a drifting grating. This measure classifies V1 cells as simple or complex (Skottun et al. 1991).

*9*) Variability. The Fano factor: the ratio of the variance of response to repeated presentations of the same stimulus, divided by the mean response, measured in 100-ms bins.

*10*) Signal power. The proportion of the total variance of the neuron's response attributable to the signal (Sahani and Linden 2003).

*11*) Burstiness. The proportion of interspike intervals shorter than 30 ms. A threshold of 30 ms was chosen because it is shorter than our briefest stimuli, flashed gratings (∼32 ms).

The first two factors are defined for pairs of neurons, whereas the remaining ones depend on the individual neurons in the pair. For these single-neuron factors, the corresponding pairwise factor was taken to be the geometric mean. The arithmetic mean yielded similar results, except in the case of firing rate, where the geometric mean was clearly superior in predicting noise correlations.

#### The nonlinear additive model.

To study the joint effect of these factors on noise correlations, we characterized their role within a single composite model. We employed a nonlinear additive model, in which the functional dependence of noise correlation on any single factor was unconstrained, but the influence of different factors summed. As the overall scale of each single-factor function was free to vary, this additive second stage was equal in generality to a full weighted linear combination. We estimated the functional influence of each factor using an iterative procedure (“backfitting”; Hastie and Tibshirani 1990). At each iteration, residuals were calculated between the measured noise correlations and the prediction of the model using all but one input factor. The influence function of the one factor left out was fit to these residuals with a smoothness constraint imposed by using Gaussian process regression (Williams 1998). The updated function was combined with the others in the model, and the process repeated, this time leaving out a different factor. These iterations continued until convergence.

We measured the contribution of each factor within the model using the proportion by which the variance explained by the full model decreased when that factor was excluded. That is, if the variance explained by the full model with all 11 factors is *V*_full_, and that by a reduced 10-factor model from which the *i*th factor has been excluded is *V*_−*i*_, then the proportional contribution *C*_*i*_ of the *i*th factor was defined to be:

$$C_{i}=\frac{V_{\text{full}}-V_{-i}}{V_{\text{full}}}$$

This measure was conservative in that *V*_−*i*_ included any variance that could be explained by other factors that covaried with the *i*th factor. Indeed, variance in noise correlation that could be explained by more than one factor would not be attributed to any of them, and so the sum of *C*_*i*_ over all factors was <1.

## RESULTS

We recorded from 843 neurons in area V1 of seven anesthetized cats, in response to four types of stimuli: natural movies, flashed gratings, drifting gratings, and blank (gray) screens (Fig. 1*A*). Neural signals were recorded using a 96-channel silicon Utah probe, and spike sorting was performed through clustering in the space of principal components, leading to well-isolated single neurons (Fig. 1*B*).

These neurons differed in the width of their averaged spike waveform. This measure, defined as the time between the peak and trough, distinguished two groups of neurons, with narrow and broad spikes (Fig. 1, *C* and *D*). The former are likely to include many fast-spiking interneurons but also a large number of excitatory cells, which in cat cortex can have thin spikes (Nowak et al. 2003).

### 

#### Effect of time scale.

We measured the noise correlations between the 22,705 pairs of neurons in this dataset and found them to be generally positive (Fig. 1, *E–G*). The average correlation, measured using 100-ms bins, was small but significantly different from zero (ρ_μ_ = 0.03 ± 0.0001). The distribution was asymmetric, with a weak negative tail and a heavy positive tail (Fig. 1*E*).

The size of correlations depended critically on the timescale at which they were measured (Smith and Kohn 2008) (Fig. 1*F*). We generally took spike counts in Δ = 100-ms bins. Using shorter or longer time scales profoundly altered correlations (Fig. 1*F*). With shorter bins (Δ = 20 ms) correlations were confined to a narrow range between −0.025 and 0.1. At longer timescales (Δ = 500 ms), this range expanded to −0.1 and 0.3 (Fig. 1*F*). Average noise correlations increased by at least an order of magnitude with increasing timescale (Fig. 1*H*). These features were shared by measurements obtained in all four stimulus conditions, except that noise correlations tended to be higher for flashed gratings and natural movies (Fig. 1, *F* and *H*, cyan and green). As we will see, these differences across stimuli should be interpreted in the context of other factors, such as the different firing rates evoked by those stimuli.

#### Eleven potential factors.

For each pair of neurons, we calculated 11 factors that could play a role in determining pairwise correlations. The first two factors were cortical distance and tuning distance, which are defined for pairs of neurons. The remaining nine factors were firing rate, spike width, spike isolation, tuning width, latency, linearity, variability, signal power, and burstiness (see materials and methods for definitions). These factors are defined for single neurons, and the corresponding pairwise factor was taken to be the geometric mean.

**Fig. 2.**
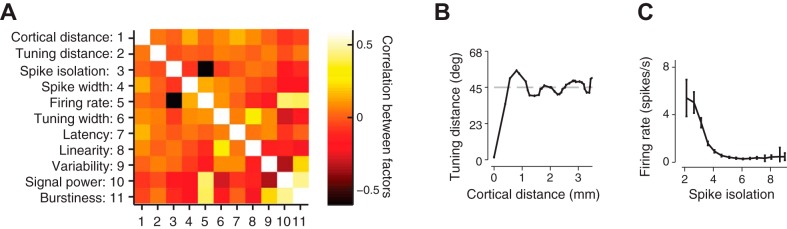
Relationships between the 11 factors that we considered as potentially affecting noise correlations. *A*: correlations between each of the 11 factors. *B*: average difference in preferred orientation between pairs of neurons as a function of their distance. Due to the orientation preference maps in cat V1, pairs of neurons at the same electrode (Δ*x* = 0 mm) had the same orientation preference (Δθ = 0°). *C*: average firing rate of neurons as a function of spike isolation. For d′ > 4 the relationship is flat, suggesting that this is a good threshold for single units.

Because of physiological and experimental constraints, however, these 11 factors were not independent of each other (Fig. 2*A*). For instance, cortical distance and tuning distance showed only weak correlation (Fig. 2*A*) but nevertheless had a clearly structured interdependence, with a spatial periodicity of ∼1.3 mm (Fig. 2*B*). This periodicity presumably reflected the spacing of orientation columns on the cortical surface (Hubel and Weisel 1962). We also observed that firing rate and spike isolation were strongly anticorrelated (ρ = −0.4; Fig. 2, *A* and *C*), most likely because less well isolated units with small d′ fell close to the multiunit noise cluster (Fig. 1*B*) and therefore contained more false-positive spikes, artifactually boosting their apparent firing rates. The leveling of the curve at around d′ = 4 suggests that this value might provide a good criterion for single-unit isolation (Fig. 2*C*).

We next explore the impact on pairwise correlations on the first 5 of these 11 factors, which turn out to be the most important ones. Then, we present a means to evaluate the impact of all 11 factors in combination.

#### Effect of firing rate.

**Fig. 3.**
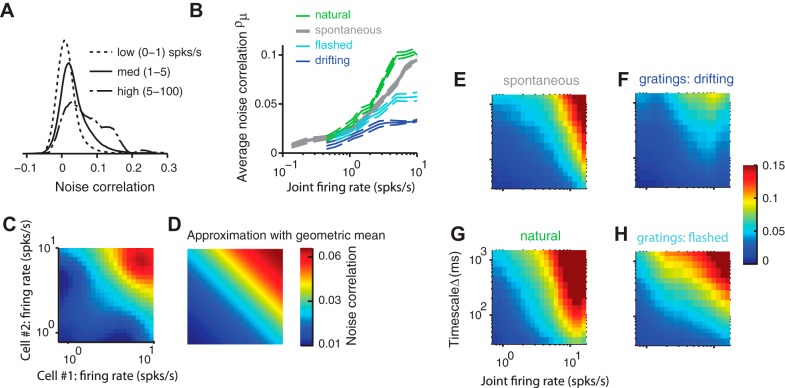
Effect of firing rate on noise correlations. *A*: distribution of noise correlations for 3 ranges of joint firing rate. *B*: average noise correlations grow with joint firing rate. *C*: dependence of noise correlations on the firing rate of each neuron in the pair. The average over all pairs is shown. *D*: taking the geometric mean of the pair's firing rates and fitting an exponential form provides a reasonable approximation. The dependence of noise correlations on firing rate can therefore be reduced to a 1-dimensional function. Note that axes are log scaled. *E–H*: noise correlations as a function of timescale and joint firing rate in each stimulus condition.

Noise correlations markedly increased with firing rate (Fig. 3, *A* and *B*). We defined the joint firing rate of a pair of neurons as the geometric mean of their average firing rates (Bair et al. 2001; de la Rocha et al. 2007; Shea-Brown et al. 2008). As joint firing rate increased, the distribution of correlations changed similarly to the effect of increasing timescale: the positive tail became heavier while the negative tail remained unchanged (Fig. 3*A*). Therefore, increasing joint firing rate markedly increased the average correlation (Fig. 3*B*).

This increase was unrelated to the purely mathematical effect due to the binary nature of spiking. This binary nature determines hard bounds on correlations, which grow with firing rate (Dorn and Ringach 2003). We computed spike-count versions of those bounds numerically given the firing rates in our data and found that the measured noise correlations were much closer to zero than these bounds (not shown).

Furthermore, the increase of correlations with firing rate depended on stimulus type. Average noise correlations increased roughly proportionally to the logarithm of joint firing rate, with the slope of this proportionality depending on stimulus type (Fig. 3*B*). At high firing rates, average noise correlations were three times larger in the responses to natural stimuli (Fig. 3*B*, green) as in those to drifting gratings (blue). The higher correlations observed with flashed gratings (Fig. 1*H*), therefore, are explained by higher firing rates elicited by these stimuli.

This measure of joint firing rate, the geometric mean, provided a reasonable summary of the effects of the individual firing rates on noise correlations (Fig. 3, *C* and *D*). The function mapping individual firing rates to average noise correlations was estimated from the data and increased with firing rates in an orderly fashion (Fig. 3*C*). This two-dimensional function was well approximated by a function taking a single input, the geometric mean of the two firing rates (Fig. 3*D*). This model explained 83% of the variance of the estimated function in Fig. 3*C*. Using the arithmetic mean resulted in a larger fitting error, accounting for only 48% of the variance (not shown). The dependence of average noise correlations on geometric mean firing rates is also in agreement with theoretical results derived from integrate-and-fire neurons (Shea-Brown et al. 2007). For subsequent analysis, we therefore used the joint firing rate of a pair defined by the geometric mean.

Noise correlations increased with firing rate at all timescales; at high joint firing rates and long timescales, they could be almost 50 times stronger than at low joint firing rates and short timescales (Fig. 3, *E–H*). The details of this effect again seemed to depend on stimulus type. At any given timescale and firing rate, average noise correlations were particularly weak in responses to drifting gratings (Fig. 3*F*). This was due to a combined effect of both weaker positive noise correlations and stronger negative noise correlations at that firing rate (Fig. 1*G*). At fast timescales, highly active cell pairs were more correlated during natural movie stimulation and spontaneous activity (Fig. 3, *E* and *G*) than during stimulation with artificial stimuli (Fig. 3, *F* and *H*).

Because firing rate exerts such a strong influence on noise correlations, in most of the remaining analyses we discounted this effect, so that we could examine the impact of other variables. Specifically, we normalized the noise correlations measured for a set of neuronal pairs by the average noise correlation that would be expected for those pairs based solely on their joint firing rate, rather than just dividing by the joint firing rates alone (Bair et al. 2001; Smith and Kohn 2008).

#### Effect of cortical distance and tuning distance.

**Fig. 4.**
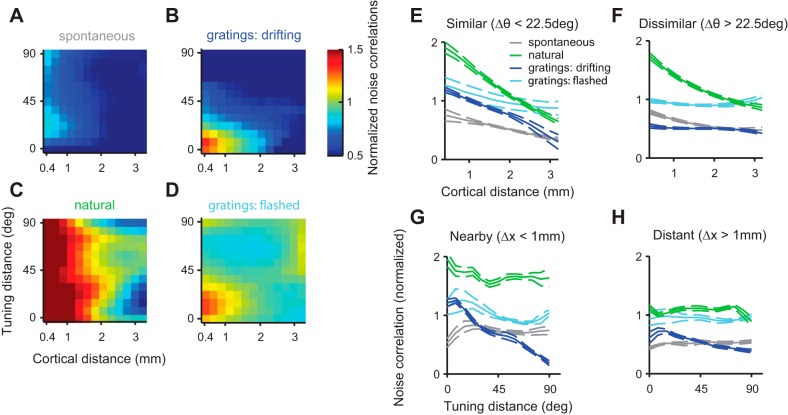
Effect of cortical distance and tuning distance on noise correlations. *A–D*: dependence of noise correlations on cortical distance and tuning distance (difference in preferred orientation between the pair). Each panel corresponds to a stimulus type, and each point represents the average noise correlation of multiple pairs divided by the noise correlation predicted from the pairs' firing rates only (Smith and Kohn 2008). *E* and *F*: dependence of noise correlations on distance in space for similarly (*E*) and dissimilarly (*F*) tuned cells. *G* and *H*: dependence of noise correlations on tuning distance for nearby (*G*) and distant (*H*) cell pairs.

Previous studies of noise correlations in visual cortex have investigated their dependence on cortical distance and on tuning distance, i.e., the difference in the neurons' preferred orientations (Toyama et al. 1981b; Ts'o et al. 1986; Smith and Kohn 2008). The common finding is that noise correlations (or related measures) decrease on average as cortical distance or tuning distance increase (Ko et al. 2011). The advent of high-count multielectrode recording techniques has allowed detailed two-dimensional maps to be built, showing how noise correlations vary with these two factors in monkey V1 (Smith and Kohn 2008). We sought to measure similar maps in cat V1 and to ask whether their structure depended on stimulus type and timescale (Fig. 4).

To compare our analysis to the findings reported in monkey V1 (Smith and Kohn 2008), we first examined the normalized noise correlations in response to drifting gratings (Fig. 4*B*, normalized for firing rates of pair). Similar to results seen in monkey V1, the normalized average noise correlation evoked by drifting gratings dropped sharply with cortical distance and with tuning distance (Fig. 4*B*). The drop due to cortical distance was particularly marked for pairs with similar orientation tuning (Fig. 4*E*, blue), and the drop due to tuning distance was particularly marked for pairs of nearby cells (Fig. 4*G*, blue).

During spontaneous activity, normalized pairwise correlations tended to be weaker (Fig. 4*A*) and less dependent on either cortical distance or tuning distance (Fig. 4, *E* and *G*, gray). Surprisingly, the pairs showing the highest average noise correlations were not those having the same orientation tuning at Δθ = 0° but rather those having slightly different orientation preferences (Δθ = 15°) (Fig. 4, *A* and *G*, grey line). This trend was small but significant (*P* < 0.001, random permutation test, *N* = 1,000 samples). A similar effect might be glimpsed in the pairwise correlations measured in the responses to flashed gratings (Fig. 4, *D* and *G*, cyan line), although here it was not significant (*P* = 0.14, random permutation test, *N* = 1,000 samples).

Finally, in responses to natural stimuli, normalized pairwise correlations were large for nearby cells (Fig. 4*C*) but showed only a weak dependence on tuning distance (Fig. 4, *G* and *H*, green). However, the overall modulation of noise correlations by cortical distance was stronger than in all other conditions (Fig. 4, *E* and *F*, green). Cortical distance still modulated correlations strongly for pairs with large tuning distance during natural viewing conditions but not during any other stimulus condition (Fig. 4*F*). Thus, under natural stimulation, correlations are very strongly modulated by distance in space but not by difference in preferred orientation.

#### Effect of spike width.

**Fig. 5.**
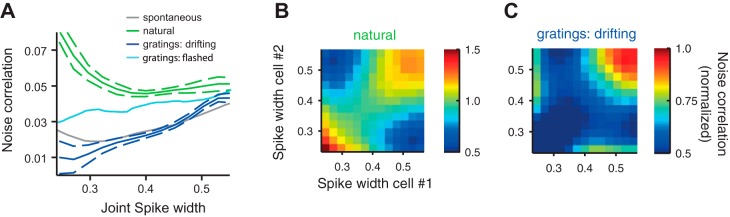
Effect of spike width on noise correlations. *A*: dependence of noise correlations on joint spike width (the geometric mean of the spike widths of the 2 neurons). Error bars are only shown for 2 conditions for visual purposes but are comparable across conditions. *B*: dependence of noise correlations as a function of spike width of each cell in a pair, during presentation of natural stimuli. *C*: same as in *B* during presentation of drifting gratings.

Having observed that cells differ in spike width (Fig. 1, *C* and *D*), we next asked whether spike width affected noise correlations. We first combined the spike widths of two neurons in a pair by taking the geometric mean (or “joint spike width”). In three of the four stimulus conditions, noise correlations increased monotonically with joint spike width (Fig. 5*A*), indicating that broad-spiking cells tended to be better correlated within their group than were narrow spiking cells. In the responses to natural movies, however, correlations did not show this monotonic relationship (Fig. 5*A*, green line). Here, jointly narrow spiking pairs were far more strongly correlated than during any other stimulus condition and, indeed, more strongly correlated than jointly broad spiking pairs. Furthermore, narrow spiking cells were significantly more correlated with each other than with broad spiking cells (Fig. 5*A*, green line, trough; *P* = 0.0021, random permutation test; *N* = 1,000 samples).

To understand these effects, we examined how normalized noise correlations depended on the spike width of each neuron in the pair (Fig. 5, *B* and *C*). In responses to natural stimuli there was a clear bipartite correlation structure (Fig. 5*B*). Neurons correlated strongly with other neurons that had similar spike shapes but relatively weakly with neurons in the other class. In response to drifting gratings, broad spiking cells were more correlated within their class than narrow spiking cells (Fig. 5*C*). The other conditions appeared similar. These observations also held for raw noise correlations, which were not corrected for the effect of firing rate (not shown).

These results, therefore, reveal added functional significance to the clustering of V1 based on spike width: the variability of the cells in each cluster is preferentially correlated with that of other cells in the same cluster. This is the case for the broad-spiking cells under all stimulus conditions, and in responses to natural stimuli it is also the case for narrow-spiking cells.

#### Effect of spike isolation.

**Fig. 6.**
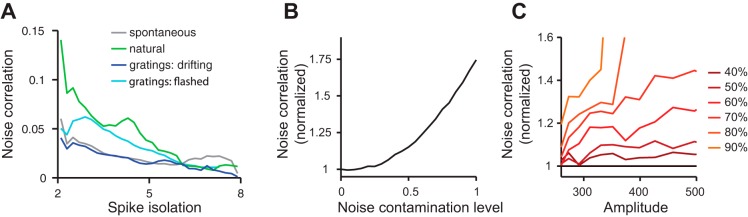
Effect of spike isolation on noise correlations. *A*: dependence of noise correlations on spike isolation. *B*: modulation of noise correlations by varying the multiunit (“noise”) contamination level of single units. *C*: modulation of noise correlations as a function of spike amplitude at different contamination levels with multiunit spikes. Note that at high contamination levels (>70%), the full range of amplitudes cannot be sampled due to the small average amplitude of multiunit spikes.

Next, we considered whether noise correlations depend on spike isolation (Fig. 6). Consistent with an earlier proposal (Ecker et al. 2010), we found that this factor had a significant effect. Noise correlations decreased markedly with the geometric mean of the spike isolation of the two neurons in a pair (Fig. 6*A*). Spike isolation was measured as the distance (d′) of the spikes of a neuron from the multiunit noise cluster. Neurons whose spikes were well isolated showed lower average noise correlations with each other than with neurons whose spikes were more poorly isolated. This effect was particularly strong for less well-isolated neurons.

A simple explanation for this observation might be that the spike sorting procedure picks up an increasing number of false positives, as the single unit cluster gets closer to the multiunit noise cluster. These false positives would be more correlated with each other: they cross the detection threshold precisely because they are composed of many small multiunit spikes potentially riding on top of global modulations of activity, which span several electrodes. To superimpose and cross threshold, they must occur nearby in time, i.e., be correlated.

To test if this explanation is plausible, we contaminated our dataset of well-isolated single units (d′ > 4) with increasing levels of spikes from the multiunit noise cluster, while keeping the total numbers of spikes (and therefore the firing rates) constant (Fig. 6*B*). For example, at a contamination level of 10%, we substituted 10% of all single unit spikes with randomly sampled spike times from the multiunit noise cluster and then recomputed all noise correlation coefficients. At a contamination level of 100%, all single unit spikes were substituted by multiunit noise events. To prevent the spikes of one neuron from swapping with spikes from the same neuron, we only considered noise correlations between neurons located on different electrodes. As the single units were contaminated with increasing levels of multiunit spike events, noise correlations increased markedly, by up to a factor of 1.75 (Fig. 6*B*). This result suggests that spike sorting may indeed be responsible for the dependence of noise correlations on spike isolation.

Another possible explanation for the dependence of noise correlations on spike isolation is that it is physiological. Spike isolation covaries with several features of the spike waveform, most notably its amplitude. Perhaps neurons with large spikes show lower pairwise correlations than neurons with small spikes? To test this hypothesis, we repeated our contamination procedure, this time restricting the pool of spikes in the multiunit cluster to match the amplitude of the spikes of the single unit. At low to medium contamination levels (<50%), there was no systematic increase of correlations with spike amplitude (Fig. 6*C*). Importantly, for a given fixed amplitude, noise correlations were stronger with increasing contamination levels (Fig. 6*C*). We conclude that the dependence of noise correlations on spike isolation is due to the relationship between spike isolation and the artifacts of spike sorting and not to a relationship between spike isolation and another physiological variable.

Thus spike isolation strongly modulates noise correlations for neurons recorded on distant electrodes. This phenomenon cannot be explained by these neurons effectively exchanging spikes, as had been previously proposed (Ecker et al. 2010). Rather, we propose that less well-isolated units contain many overlapping spikes caused by synchronously firing neurons excited by widely shared modulations of activity.

#### The nonlinear additive model.

Up to now we have examined the effect of multiple factors on pairwise correlations, mostly in isolation. However, we have also seen that these factors are not independent of each other (Fig. 2, *A–C*). This interdependence makes it difficult to separate out their influence: the apparent relationships between noise correlation and each factor, or pair of factors, could have been altered by dependence on a different, but covarying, third factor. For example, a dependence of noise correlation on tuning distance could lead to an apparent relationship with cortical distance simply through the link between these two predictive factors. Similarly, an apparent dependence on firing rate could in principle just reflect the impact of spike isolation. Unfortunately, it is not possible to probe the effect of each factor while holding the others fixed, because most factors were not under experimental control.

Thus it was necessary to understand the joint effect of the factors through their role within a single composite model. This model uses all 11 factors together to predict the noise correlation between a pair of neurons, thus providing the power to isolate the estimated relationship from potentially confounding factors. The simplest such model would have been linear; however, we had no reason to expect the dependence of noise correlation on any factor to be linear, or even monotonic.

**Fig. 7.**
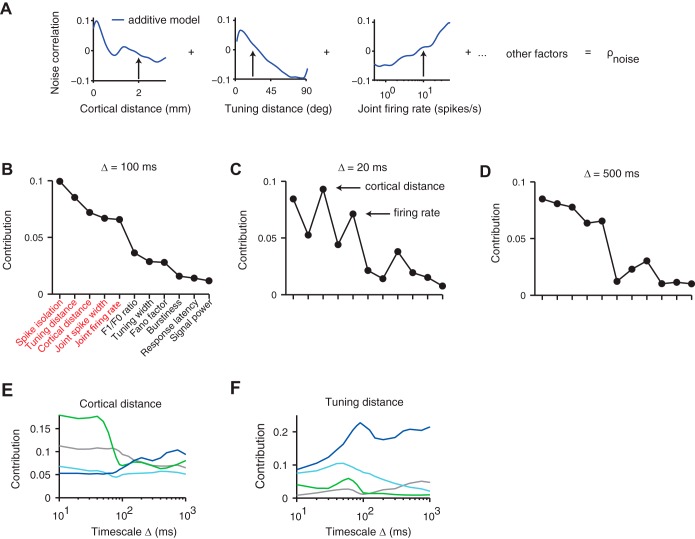
Use of a nonlinear additive model to rank the role of multiple factors in noise correlations. *A*: in the model, each pair of neurons is associated with eleven factor values. The model predicts their noise correlation by summing the function values of 11 one-dimensional functions corresponding to those factors. *B*: ranking of factors according to their contribution to noise correlations averaged over all 4 stimulus types (Δ = 100 ms). *C* and *D*: a largely similar ranking was seen at 2 different timescales (Δ = 20 ms, 500 ms). *E*: contribution of the cortical distance factor as a function of timescale for each stimulus condition. *F*: same as *E* but with tuning distance factor.

Therefore, to investigate the dependence of noise correlations on all predictive factors jointly, we used a nonlinear additive model (Fig. 7*A*). We used Gaussian process regression (Williams 1998) to fit the influence of each individual factor by a smooth, nonparametric function, which could be nonlinear and indeed nonmonotonic (Fig. 7*A*). The noise correlation of each pair was described by the sum of such functions, which is equivalent to a general linear combination. This allowed the model to be fit robustly to the available volume of data, and retained the interpretability of each individual factor function.

The model provided a good account of the dependence of noise correlations on the 11 factors, explaining 50% of the total variance during stimulation with natural stimuli and 30% during the remaining stimulus conditions. Since the total variance includes an unknown noise variance, which cannot be predicted by any model, the reported performance of the model is a lower bound on its true performance.

#### Five key factors.

Having established the validity of the nonlinear additive model, we used the model to rank each factor according to its impact. We ranked each factor according to the proportion by which the variance explained by the full model decreased when that factor was excluded (see materials and methods). This measure quantified the explainable variance uniquely attributable to each single factor, discounting any variance that could be explained in more than one way. The resulting contributions were averaged across stimulus types.

This analysis revealed five key factors that stood out (Fig. 7*B*): spike isolation, tuning distance, cortical distance, joint spike width, and joint firing rate. These five factors were noticeably more important than the remaining ones, not only at our standard timescale of Δ = 100 ms (Fig. 7*B*), but also at shorter and longer timescales (Fig. 7, *C* and *D*), although at Δ = 20 ms cortical distance and joint firing rate had a larger explanatory role. Running the *k*-means clustering algorithm yielded the same clustering into five important and six less important factors as is visually obvious (Fig. 7, *B* and *D*), with both groups having significantly different means (*P* < 1e-04, two-sample *t*-test).

These same five factors also remained most influential when data were broken down by stimulus type, although the relative importance of cortical distance and tuning distance changed markedly from one stimulus type to another, and across timescales (Fig. 7, *E* and *F*). The importance of cortical distance showed a marked transition for natural stimuli at a timescale of 100 ms (Fig. 7*E*). Up to that timescale, the contribution of cortical distance is much larger than for other stimulus types (Fig. 7*E*, green). The contribution of tuning distance, conversely, was strongest in responses to gratings (Fig. 7*F*) and peaked at the characteristic time of each stimulus: long time scales for drifting gratings, which retained a single orientation for seconds (Fig. 7*F*, blue), and short time scales for flashed gratings, which changed orientation every 33 ms (Fig. 7*F*, cyan).

**Fig. 8.**
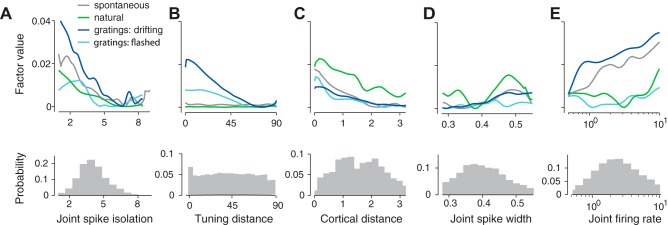
Dependence of the five key components on the respective factors. *A–E*: shape of the 5 key factors estimated by the nonlinear additive model in each stimulus condition. Density plots of measured factor values in the neural populations in V1 are underneath each corresponding factor.

The dependence on each of the five key predictive factors was generally monotonic (Fig. 8): noise correlations tended to decrease with increasing cortical distance, tuning distance, and spike isolation and to increase for broader-spiking and higher firing rate pairs. An exception was the dependence on joint spike width during presentation of natural stimuli, where the strong within-group noise correlations of jointly narrow and jointly broad spiking cell pairs (Fig. 5) were reflected in a bimodal functional dependence within the model (Fig. 8*D*, green line). This bimodality confirms that cells are more noise correlated within their groups than across groups (Figs. 5*B* and 8*D*).

The general features of these model functions are consistent with the curves of average noise correlation computed previously as a function of each individual factor. However, the model untangles the correlations between factors and therefore produces some observations that correct those gathered from simply averaging across correlation curves. For example, when joint firing rate was considered alone, it seemed to play the strongest role during presentation of natural stimuli (Fig. 3*B*, green). When instead it was considered during presentation of drifting gratings and spontaneous activity, it actually played a larger role in responses to drifting gratings and in spontaneous activity (Fig. 8*E*, blue and gray). A similar effect was seen for spike isolation: when considered alone, it seemed to play the largest role in responses to natural stimuli (Fig. 6*A*), but in fact its contribution is largest in responses to drifting gratings (Fig. 8*A*).

The shapes of most model functions remained relatively constant when predicting noise correlations at different timescales (not shown). In other words, the model functions measured at short and long timescales looked similar, up to a multiplicative factor, to those measured at a timescale of 100 ms (Fig. 8). The multiplicative factor accounts for the fact that noise correlations increase with timescale (Fig. 1*H*). Thus the effect of timescale on most of the model functions can be accounted for by normalization with the average noise correlation at that timescale. The only exceptions to this rule were cortical distance and tuning distance, whose functions changed across timescale in a manner that could not simply be described by a multiplicative factor. Rather, the changes of their shapes mirrored the behavior of their respective contributions (Fig. 7, *E* and *F*).

These observations indicate that our nonlinear additive model can disambiguate the contributions of different factors to noise correlations. With a few exceptions, these contributions are generally monotonic and independent of stimulus type and timescale of analysis.

## DISCUSSION

Based on a large dataset of 22,705 neuronal pairs, we have identified five key factors that influence the strength of noise correlations. The five key factors relating to pairwise noise correlation are, in order of importance, as follows: spike isolation, cortical distance, tuning distance, spike width, and firing rate. We were able to identify and rank these as the most influential of 11 factors we considered by using a nonlinear additive model to untangle the contributions of individual factors even though the factors did not vary independently from pair to pair.

The most important factor that we identified, the quality of spike isolation, had been proposed to play a role in determining correlations only for neuron pairs measured at single electrodes (Ecker et al. 2010). Here, we showed that it plays a crucial role even when the neurons in the pair are recorded from different electrodes. We performed simulations in which spikes were intentionally mislabeled and showed that the results are due to the mislabeling of spikes belonging to the activity of many neurons whose spike waveforms are too small to resolve (multiunit activity) compared with spikes belonging to a well-isolated neuron (single-unit activity). The correlations between multiunit activity, in turn, are well-known to be larger than those of single-unit activity (Bedenbaugh and Gerstein 1997).

The effects of spike isolation on correlations across electrodes were larger than anticipated based on previous simulations. A previous study investigated the impact of spike sorting errors by pooling over weakly correlated single unit clusters (Cohen and Kohn 2011). It concluded that the effect of these errors on noise correlations was relatively weak and was unlikely to strongly inflate noise correlation estimates. In our dataset, instead, we found that the effect of spike isolation can indeed be very strong, especially when the single unit cluster is close to the multiunit noise cluster (d′ < 4). We suggest that strongly correlated spikes, which individually are too weak to cross threshold, superimpose to form events that are classified as noise due to their shape but that are, by definition, strongly temporally correlated with each other. Finally, since both the dependency of firing rate on spike isolation and the model functions of the spike isolation factor are flat for d′ > 4, we propose this value as a good threshold for well-isolated neurons.

The next two main factors determining pairwise correlations, cortical distance and tuning distance, have long been known to affect correlations (Toyama et al. 1981a; Ts'o et al. 1986; Smith and Kohn 2008). The common finding is that noise correlations decrease on average as cortical distance or tuning distance increase (Ko et al. 2011); our data confirm that tuning similarity (in our case, orientation) and separation in cortical space play a strong role. However, these two factors are sensitive to stimulus type and timescale. At fast timescales (Δ = 20 ms), cortical distance is the most important factor overall. It is particularly dominant during presentation of natural stimuli but drops sharply in importance at timescales slower than 100 ms (Δ > 100 ms). Tuning distance, on the other hand, is the determining factor during presentation of gratings, especially drifting gratings. There, its importance increases steadily with timescale, whereas it peaks at a characteristic timescale during flashed gratings and then declines. Remarkably, during presentation of natural stimuli, tuning distance has a very small influence on noise correlations.

The fourth factor in order of importance was spike width, and as far as we know it had not been previously proposed to play a role in noise correlations. We generally found the strongest correlations to be between broad spiking cells, with pairs of narrow spiking cells being significantly less correlated. These differences did not result from differences in firing rates alone, as the normalized noise correlations showed the same structure. An exception to this general pattern occurs during presentation of natural stimuli: noise correlations between pairs of narrow spiking cells then increased in strength to levels even beyond those observed between broad spiking cells. Noise correlations between narrow and broad spiking cells remained low, as was the case in all other stimulus conditions. The origins of this phenomenon are at the moment unclear. It is unlikely to reflect a simple distinction between inhibitory and excitatory cells, because in cat cortex these cell classes are not neatly distinguished by spike width (Nowak et al. 2003).

Finally, the fifth factor, firing rate, has been widely believed to play a role in determining correlation. Higher firing rates have been shown to lead to higher correlations in simulations (Dorn and Ringach 2003; de la Rocha et al. 2007; Shea-Brown et al. 2008), and in vitro (de la Rocha et al. 2007). However, studies in vivo have been less consistent, with some reporting firing rate to increase correlations (Bair et al. 2001; Cohen and Maunsell 2009; Cohen and Kohn 2011), others finding only weak dependence (Ecker et al. 2010) and yet others finding no dependence (Kohn and Smith 2005). Our data support a strong dependence on firing rates and indicate that the growth of correlations with firing rate is best described by a saturating function taking the geometric mean of the cell pair's firing rate as an argument. Therefore, a simple way to normalize noise correlations by firing rate is to divide correlations by the prediction given by this function.

These five factors dominated the other six that we considered in this study, which were as follows: width of the orientation tuning curves, latency of response, linearity of response, variability of response (Fano factor), signal power, and burstiness (the propensity of the cell to fire spikes separated by <30 ms). Although the contribution of these additional factors was nonzero, they all clearly fell into a second, less influential group when comparing their importance with the five key factors. This result was robust across stimulus types and timescale.

However, our data did not allow us to consider some further factors that are likely to shape noise correlation. Two such candidates are the cortical depth of the layer (Hansen et al. 2012; Smith et al. 2013) and cell type. Because of electrode length and neuron numbers, most of the cells recorded in our dataset are likely to be pyramidal cells in layer 2/3 or layer 4. It is possible that had we been able to distinguish between layers or between cell types (e.g., excitatory vs. inhibitory), we would have seen systematic differences in correlation. The strong distinction between correlations seen among broad-spiking cells and narrow-spiking cells that we have observed points in this direction but is difficult to interpret directly because in cat, unlike in mouse, not all narrow spiking cells are inhibitory (Nowak et al. 2003).

Another global factor that surely influences noise correlations is cortical state, which can vary with the depth of anesthesia and in wakefulness (Ecker et al. 2014; Schölvinck et al 2015). Since noise correlations by definition measure correlated variability induced by input that does not repeat across trials, any unobserved modulation in excitability shared by neurons in cortex will contribute to the final measure of noise correlations. Indeed, the lowest correlations are seen in conditions in which the cortex is desynchronized (Renart et al. 2010).

Pairwise correlations constitute a powerful data set on which one can test lower dimensional representations of population activity, and it is thus essential to understand their dependence on stimuli, neurons, responses, and analyses. The list of five key factors that we provided is likely to be incomplete, and a better list is likely to include cortical layer, cell type, and one or more measures of cortical state. Still, our analyses, together with the existing knowledge of the effects of these additional factors, provide a broad picture of the factors that affect noise correlations. As we have seen, average noise correlations can vary across more than two orders of magnitude, depending on stimulus type, the timescale investigated, and the distribution of factor values recorded. It is therefore critical to carefully consider all of these factors when comparing the strength of noise correlations across studies. Indeed, variations in these factors are likely to be responsible for the apparent discrepancy between measures performed in different studies.

## GRANTS

This work was supported by the COMPLeX doctoral program, the Gatsby Charitable Foundation, the Wellcome Trust, and the European Research Council (project CORTEX). M. Carandini holds the GlaxoSmithKline/Fight for Sight Chair in Visual Neuroscience.

## DISCLOSURES

No conflicts of interest, financial or otherwise, are declared by the author(s).

## AUTHOR CONTRIBUTIONS

Author contributions: D.P.A.S., M.S., and M.C. conception and design of research; D.P.A.S. performed experiments; D.P.A.S., M.S., and M.C. interpreted results of experiments; D.P.A.S., M.S., and M.C. edited and revised manuscript; D.P.A.S., M.S., and M.C. approved final version of manuscript; D.P.A.S. analyzed data; D.P.A.S. and M.C. prepared figures; D.P.A.S. and M.C. drafted manuscript.

## Appendix

The following derivation pertains to the unbiased estimators for noise variance and covariance we adopted (Sahani and Linden 2003). The unbiased signal covariance matrix between a pair of neurons *x* and *y* with spike counts *r*_*x*_^(*n*)^, *r*_*y*_^(*n*)^ and trial-averaged responses μ_*x*_, μ_*y*_ was computed as

$$C_{xy}^{S}=\frac{1}{N-1}\left(N\;\mathrm{cov}\left(\mu_{x},\mu_{y}\right)-\frac{1}{N}\overset{N}{\underset{n=1}{\sum }}\mathrm{cov}\left(r_{x}^{\left(n\right)},r_{y}^{\left(n\right)}\right)\right)$$

where *n* denotes trial number and *N* the total number of trials. This measure is also related to the area under the cross-covariogram, which was used in some previous studies (Smith and Kohn 2008). Specifically, the zero-lag noise covariance in bins of width Δ between spike trains *X* and *Y* is identical to the integral of their cross-covariogram under a zero-centered triangular window with full width 2/Δ:

$$\begin{array}{cc} C_{xy}\left(0\right)= & \frac{1}{K}\overset{K-1}{\underset{k=0}{\sum }}\left(X_{k}-\overset{¯}{X}\right)\left(Y_{k}-\overset{¯}{Y}\right)\\ = & \frac{1}{K}\overset{K-1}{\underset{k=0}{\sum }}\left(\left(\overset{\left(k+1\right)\Delta -1}{\underset{t=k\Delta }{\sum }}x_{t}\right)-\Delta \overset{¯}{x}\right)\left(\left(\overset{\left(k+1\right)\Delta -1}{\underset{t^{\prime }=k\Delta }{\sum }}y_{t}\right)-\Delta \overset{¯}{y}\right)\\ = & \frac{1}{K}\overset{K-1}{\underset{k=0}{\sum }}\left(\overset{\left(k+1\right)\Delta -1}{\underset{t=k\Delta }{\sum }}\left(x_{t}-\overset{¯}{x}\right)\right)\left(\overset{\left(k+1\right)\Delta -t}{\underset{\tau =-\left(t-k\Delta \right)}{\sum }}\left(y_{t+\tau }-\overset{¯}{y}\right)\right)\\ \overset{E}{\overset{︷}{=}} & \overset{\Delta }{\underset{\tau =-\Delta }{\sum }}\frac{\left(\Delta -\left|\tau \right|\right)}{\Delta }\sigma_{xy}\left(\tau \right) \end{array}$$

with *K* bins, spike counts *X*_*k*_, *Y*_*k*_ in *k*th bin, *X̄*, *Ȳ* average spike counts, *x*_*t*_, *y*_*t*_ binary spikes. The last equality means “equal in expectation,” assuming a stationary process.
